# Salience Models: A Computational Cognitive Neuroscience Review

**DOI:** 10.3390/vision3040056

**Published:** 2019-10-25

**Authors:** Sofia Krasovskaya, W. Joseph MacInnes

**Affiliations:** 1 Vision Modelling Laboratory, Faculty of Social Science, National Research University Higher School of Economics, 101000 Moscow, Russia; 2 School of Psychology, National Research University Higher School of Economics, 101000 Moscow, Russia

**Keywords:** salience, computational modelling, deep learning, Itti and Koch

## Abstract

The seminal model by Laurent Itti and Cristoph Koch demonstrated that we can compute the entire flow of visual processing from input to resulting fixations. Despite many replications and follow-ups, few have matched the impact of the original model—so what made this model so groundbreaking? We have selected five key contributions that distinguish the original salience model by Itti and Koch; namely, its contribution to our theoretical, neural, and computational understanding of visual processing, as well as the spatial and temporal predictions for fixation distributions. During the last 20 years, advances in the field have brought up various techniques and approaches to salience modelling, many of which tried to improve or add to the initial Itti and Koch model. One of the most recent trends has been to adopt the computational power of deep learning neural networks; however, this has also shifted their primary focus to spatial classification. We present a review of recent approaches to modelling salience, starting from direct variations of the Itti and Koch salience model to sophisticated deep-learning architectures, and discuss the models from the point of view of their contribution to computational cognitive neuroscience.

## 1. Introduction

Roughly two decades ago, Laurent Itti and Cristoph Koch presented their model of bottom-up visual attention [1] based on the cognitive theoretical foundations of human visual attention. The model subsequently became a seminal work in the field, inspiring a multitude of researchers from various domains to propose their own models of visual attention. The Itti and Koch model was so influential because it combined several different aspects to reach one goal: to understand human visual processing by simulating the processing stages from scene level input to fixation selection. The model had a strong basis in cognitive theory and tried to replicate some neuronal mechanisms involved in visual attention by using many of the best approaches from computational vision available at the time. The result was a model that was to account for many of the spatial and the temporal aspects of human shifts of visual attention.

Subsequent models were numerous and improved the initial approach from the point of view of cognitive mechanisms, the biological accuracy, or simply augmented the bottom-up approach with additional semantic, object, or top-down information. Others were interested in using the general structure for application purposes such as computer vision and robotics. These critiques, improvements and augmentations of the original model have certainly added to what is now a rich literature in models of salience, and we do not intend to belittle their contribution. The fact that Itti and Koch are still being discussed, critiqued, and augmented 20 years after the original article is, in our opinion, a true sign of its importance in defining this field. We provide a review of the different approaches in modelling visual attention based on their contribution to computational cognitive neuroscience. We limit our review to models that were directly influenced by the Itti and Koch algorithm, and cover examples that model object attention, top-down influence, information theory, dual stream models, and conclude with recent advances in deep learning salience classifiers. We also include other methods of achieving the main goal: modelling image salience and the way it results in shifts of attention. The main questions this review tries to address are what contribution did these models make in our goal to explain human visual attention using computer simulations, and what directions are available for the next generation of models?

In this review, we approach the salience problem from a computational cognitive neuroscience perspective, which implies a combination of theoretical and methodological concepts from several domains. Such a perspective suggests that the perfect salience model should be based on a strong theoretical foundation, model the neurobiological processes underlying visual saliency, use explicit computational tools as a means of modelling these processes, and be generative by taking both spatial and temporal predictions of visual salience into account.

Studies of human visual salience have led to the creation of hundreds of computationally valid models, however, most of these models do not focus on all of the abovementioned components of salience simultaneously. We understand that with so many parameters to account for, like combining a broad cognitive theoretical scope and a focused precise neural approach, it is almost impossible to avoid trade-offs. Nevertheless, we will cover models that attempt to address at least several of the abovementioned aspects of visual salience simultaneously.

## 2. The Itti and Koch Model: Why Is It Seminal

**Figure 1:**
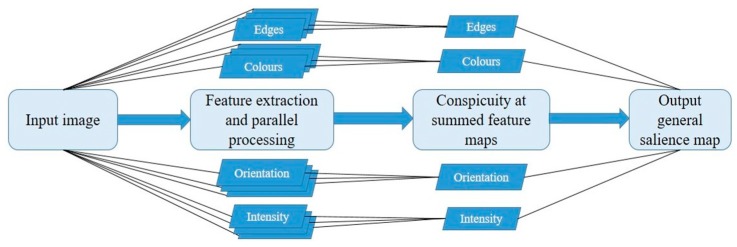
This figure is a demonstration of the salience computation algorithm present in many existing models meant to provide a general idea of the main steps required for bottom-up salience computation. In the current example, basic features like edges, colour, orientation and intensity are included, though the initial Itti and Koch model focused on three main channels: intensity, orientation and colour. Many implementations of the model take into account other features like edges, motion, junctions, shades, etc. Some implementations also include an additional temporal stage that computes fixation locations with a mechanism like inhibition of return (IOR) to prevent fixations at previously attended locations. The main steps present in the figure are as follows: (1) A model receives an input image; (2) features of the image are extracted and organized in a hierarchical manner, with separate feature channels consisting of several levels based on the scale of processing (usually features are downscaled). The features are extracted to individual feature maps using parallel processing, which means that separate feature channels are analysed simultaneously in parallel; (3) the feature maps extracted at every level are merged into one general feature map organized per feature, with the most conspicuous locations reflected on the maps; and (4) the resulting feature maps are combined into a single map of conspicuity, which is the output of the model in the form of a so-called salience map.

One of the most significant works in the field of visual attention was the study of bottom-up attention by Christof Koch and Shimon Ullman [2] that attempted to explain the underlying biological mechanisms of visual attention. The result served as a foundation for the renowned salience model, the first implementation of which was realized in 1996 [3]. The model was later refined to the version now deemed classic [1,4,5]. A schematic of these general steps for salience computation is demonstrated in Figure 1.

According to the model, the basic features of a visual scene were organized into feature maps of colour, orientation and intensity based on eight scales of linear filtering. The feature maps were then normalized within each feature based on centre-surround differences [6] with the eight scales allowing for different receptive field sizes. Specifically, scales with greater downsampling (surround) could be contrasted with scales of lesser downsampling (centre). The result was 42 maps (six intensity, 12 colour, 24 orientation) containing local spatial contrasts. Neurons within each feature map competed for the most salient locations based on a mechanism of lateral inhibition. The term ‘neuron’ in the model is simply a mathematical function that is activated upon reaching a certain threshold. Neurons with higher activations are more likely to win against its surrounding competitors. Unlike neurons in a deep learning neural network (for details, see Section 5), the neurons in the Itti and Koch model were not organised hierarchically but were activation function representations distributed throughout the map with the sole purpose of burst activation from salient feature input signals. The feature maps were then combined into a conspicuity map for each of the three features and normalized according to that map’s global maximum to prevent strong but common features in one modality from overriding weaker singletons in another feature modality. The conspicuity maps were finally united into a single 2D topographical map of general conspicuity or salience.

The merged salience map was then implemented as a two-dimensional array of Leaky Integrate-and-Fire (LIF) neurons (see reference [7] for a review of the LIF model) that represented the spatial topography of the input scene. A temporal winner-take-all algorithm was applied to the map in order to select a location of maximal salience as the first attended location [1,8]. This was an iterative process with subsequent fixations determined by a similar process. Previously attended high salient locations were discouraged from repeated fixations by an implementation of inhibition of return (IOR) [9,10,11]. Similar to the way biological centre-surround receptive fields work when distinguishing basic features of an object [6,12], the model distinguished salient object features from the background based on colour, orientation, and intensity in order to select a location for attention direction. The final layers combining LIF, winner-take-all (WTA), and IOR allowed the model to make predictions about temporal order and latency of fixations in addition to their spatial salience.

There are many reasons why this model has maintained relevance. It modelled the entire flow of visual information from visual input to fixation selection, it was easy to test against a variety of human experimental data, it has also garnered interest from applied vision researchers who wish to test more biologically plausible solutions for machine vision and robotics [13,14], and it had a strong theoretical basis that strived to implement our strongest theories of vision and attention. For example, the earliest processing stages were an implementation of the feature integration theory [15] with features processed pre-attentively and in parallel. Likewise, IOR was used as a search facilitator [10,16] to reduce the likelihood of repeated re-fixations of salient locations.

The model was based on biological mechanisms: centre-surround receptive fields sensitive to a specific set of features—pyramidal cells, projecting through the primary (V1), second (V2), dorsolateral (DL), and inferior temporal (IT) visual areas and serving as output pathways for the visual cortex [1], and leaky integrate-and-fire (LIF) neurons, which have strong global inhibition, where the entire population of neurons is inhibited in response to an activation of any single neuron in the population. Action potentials caused by such activation shift the focus of attention to the location where the signal is active. Finally, the model was generative and as such could simulate likely shifts of attention given any sample input. These shifts made spatial predictions that can be compared to real fixation locations during scene viewing but also temporal predictions in the order and latency of those attentional shifts. It is certainly possible for a model to contribute to our understanding of visual processing without addressing all of these areas, but models are particularly adept in overcoming the reductionism of controlled experiments and should continue to be evaluated in terms of how well they explicitly compute in terms of theory and biology in both the spatial and temporal domains.

## 3. What Is the Salience Problem?

It is impossible to process everything in our visual field simultaneously, so the notion of visual salience is used to describe how specific aspects of the visual field are selected for further processing. We define a salience map as a 2D accumulation of visuo-spatial information that includes, but is not limited to, bottom-up information from input image features. Since this definition includes the possibility of top-down influence, it could also be accurately referred to as a priority map [17], but we keep the term salience for consistancy with the original model. Like the many salience models suggest, this map provides a source for effective shifts of attention over time.

One candidate brain region responsible for mapping salience is the superior colliculus (SC), which is a layered structure in the dorsal part of the mammalian midbrain that directs behavioural responses toward specific locations in egocentric space. Activity in superficial layers of SC has been used to predict fixation locations in free scene viewing and visual search paradigms [18]. A salience map is represented as a two-dimensional array in the SC, where the field size increases with depth [19]. Another proposal for a neural map (for non-human primates) is LIP [20,21] (also reference [17], though they define their priority map as more strongly influenced by top-down attentional factors). This simple priority map may then work with FEF to tag previous locations [22] and SC as the result of the WTA [17].

To be effective, a salience map should be constructed from a combination of bottom-up and top-down information (the nature of this interaction is still a source of debate and beyond the scope of this review). The first area responsible for bottom-up visual attention after information is received on the retina is the lateral geniculate nucleus (LGN). Visual information from the LGN is sent toward V1, where it is then passed on to higher structures via two major pathways—the dorsal pathway, also known as the ’where’ pathway responsible for identifying spatial locations and detecting movements, and the ventral pathway, known as the ’what’ pathway, which deals with the identification of features and objects [23,24,25]. The dorsal pathway includes areas V1, V2, V3, MT, MST, and the posterior parietal cortex (PPC), from where projections reach the dorsolateral prefrontal cortex (dlPFC). The lateral intraparietal (LIP) area is also believed to be a part of this stream [26]. Areas comprising the ventral pathway include V1, V2, V4, the inferior temporal cortex (IT), and the ventrolateral prefrontal cortex (vlPFC) [27].

A top-down contribution includes information that is task-relevant and does not depend on simple visual salience of objects and locations [28]. Alfred Yarbus [29] demonstrated that fixation locations depended on the tasks that the participants carried out based on the directions given when studying natural scenes and the results of this study have been used to create a number of classifiers [20] and models [21,30] that predict the task carried out by observers based solely on their eye movement patterns. The evidence for task-based attention has served as basis for models of visual attention that take this factor into account [31,32]. Top-down attention is associated with a higher neuronal activation for behaviour-relevant stimuli and with neuronal inhibition for behaviour irrelevant stimuli [33]. As in bottom-up attention, such activations take place in areas of the dorsal and ventral visual streams, but if bottom-up processes mostly rely on feedforward connections and start in the lower areas, top-down attention is manifested through reciprocal feedback pathways that originate in the PPC and especially in PFC [34,35]. Several studies have also demonstrated that the fronto-parietal regions of the brain play a crucial role in attentional control [36,37,38]. Liu and colleagues [39] have highlighted the role of the intraparietal and precentral sulci in the choice of a fixation location, and there is evidence that the fronto-parietal network is active during visual search tasks [33,40,41,42]. Extensive research involving microstimulation [43], chemical inhibition [44], visual search tasks [45], and working memory and behavioral tasks [46] indicate a high level of involvement of the frontal eye fields (FEF) in top-down attention.

Such a strict division of processes into top-down or bottom-up is, of course, oversimplified [47,48], and both factors work together in order to produce a salience map that implements both top-down and bottom-up factors [1,49,50,51,52]. Wolfe and Horowitz [53] further highlighted five factors to support the idea that visual attention depends more than on just bottom-up and top-down approaches. The Itti and Koch model is primarily based on bottom-up feature processing, though it does contain a very limited option for top-down ‘priority’. The model is able to assign weights to the different bottom-up features of the image, thus changing the degree of priority of these features (such as colour intensity or orientation, for instance) in the final conspicuity map. The model does not, however, model any other top-down components such as semantics, world knowledge, volitional control, or task-dependent behaviour, a limitation which is explicitly stated by the authors themselves [5].

In the Itti and Koch model, selection of attentional shifts are handled by the LIF and WTA layers. In humans and primates, the final control of attentional shifts is regulated by several brain regions that include structures in the occipital, parietal, temporal, and frontal cortices, such as the cerebral cortex, basal ganglia, thalamus, superior colliculus, brainstem reticular formation and cerebellum [54,55,56,57]. Deeper layers of the SC are responsible for the coding of eye movements [55]. In the Itti and Koch model, the LIF spiking layer is considered a valid implementation of the two-dimensional array of the SC [54], which consists of so-called peaks of excitation that react to projections from the frontal eye fields (FEF) and supplementary eye fields (SEF) [58]. The peaks of the array follow a ‘winner-takes-all’ (WTA) strategy, competing with each other in order to reach a certain threshold that would serve as a saccade initiator. This strategy is supported by lateral inhibition, which is responsible for the control of spatial attention by the process of inhibition of all neighbouring signals except for a single peak of neurons that would reach the threshold [1,15,59].

Consequently, the creation of an artificial model of human salience is a challenging goal, especially given the rich literature on the biological mechanisms of visual attention. An effective model of salience and visual processing, however, does not need to explicitly model the functional areas involved in bottom-up or top-down processing. In fact, a full simulation is likely beyond our current ability. However, the closer a model stays to biological realism, the closer we get to understanding the conection between neural function and resulting behaviour.

## 4. Computational Salience Models

The idea to model selective attention grew alongside the cognitive revolution in psychology [60], and grew with the sophistication of the inspiring theories and with the computational power to implement them. Many models emerged alongside and after Itti and Koch, and they provided extensions and alternate implementations of the process. Although the division of models into separate classes may not be categorical, we group them based on their most vivid characteristics. Thus, we highlight the following groups of models: models directly inspired by the Itti and Koch architecture, biologically driven models, top-down models, models based on the dorsal and ventral pathways, object models, and computationally driven models.

### 4.1. Direct Variations of the Itti and Koch Model

The initial Itti and Koch model served as a foundation for many subsequent models that may share the basic structure of the original, but deviate or expand from it in some important aspects. In this subsection, we focus on such models and list their similarities and discrepancies as compared to the Itti and Koch model.

One of the first variations was called Attentional Selection for Object Recognition [61], and like the name implies, it was based on the classical Itti and Koch model but with a hierarchical component for object recognition (HMAX) [62]. While the original model was able to focus on salient regions in space, HMAX added an explicit implementation of regions as objects. The HMAX module was hierarchically organised with initial layers matching the Itti and Koch model but then used feature combination and pooling functions to combine information as it moved up through the layers.

Keeping with the focus on neural plausibility, the mid layer of the HMAX module represented composite feature cells and functionally corresponded to area V4 in the visual cortex. The activity of units in this layer were subject to an attention modulation process responsible for approximating spatial location information and representing salient objects in the visual scene. The final layer was able to recognise specific objects with tolerance to changes in scale and position. This model also used IOR to prevent refixations, but instead of being based on spatial locations like in the Itti and Koch model, inhibition was object-based [63]. Although it shared many features with other salience models, HMAX was focused on the task of object recognition rather than spatial and temporal fixation simulation.

Since many early models focused on pixel level input, they were unable to compare salience between 2D transformations such as reflection, rotation and translation. The Selective Attention as a Front End (SAFE) model [64] accounted for these transformations by working horizontally across all feature channels for each feature scale, instead of vertically within each feature channel. For a better understanding, feature channels and scale levels may be regarded as a matrix, where feature channels are columns and scale levels are rows. In the vertical approach, features are analysed by separate columns, while the horizontal approach implies feature analysis by rows that include different features located on one row of the matrix. The model used transformed variants of the same image to calculate the error of fixations for the base image to fixations for the transformed image. Processing in the model was based on pyramids of intensity, colour opponency, and edges, and used difference of Gaussians to assess salience. Fixations were represented as a location and scale value inside the salience pyramid. Theoretically, the model supported Eriksen and James’ [65] metaphor of visual attention as a zoom-lens by taking the scale of the image into account as opposed to the strict spotlight theory [66]. This resulted in an improved model for appearance-based object recognition systems that attended to the same features across different scales and geometric transformations.

As theories of attention and salience improved, so did the models that chose to implement them. Where feature integration theory allowed for preattentive processing based on basic features, Guided Search [67] proposed that attention could be guided by that preattentive information. Navalpakkam and Itti [68] created a model that used guided search to implement task-relevance, attention bias for low-level target features, target recognition based on basic features, and the creation of a task-relevant map of locations in the visual scene in addition to biased salience maps. The model had a working memory component responsible for memorizing task-relevant items and a long-term memory component used for hierarchical matching of items based on the given task. As a result, the model produced a topographic task-relevant map of locations and item relevance with regards to the task. The structure of the model used bottom-up salience [1,5] with separate feature maps in parallel fashion, but these maps underwent a process of top-down guidance via weight adjustment in order to separate task-relevant from task-irrelevant features.

The model did not incorporate a sufficient level of top-down control in order to make attentional shifts directed toward specific objects (a man’s hand) or locations (bottom-right object) as this would require a spatial relation component. Moreover, it used fixed object signatures and was not able to adapt to distances and rotations of objects in the visual scene. However, it was a good example of the interaction between attention and working memory. There is enough evidence supporting the overlap between the two constructs during task-driven actions, which facilitates the choice of information relevant to the goal [69,70]. Moreover, there is evidence of visual working memory acting as an attentional guiding mechanism in visuospatial tasks [71] and of FEF neurons responding differently to stimuli that have already been attended to [22].

In addition to cognitive theories, some models chose to borrow aspects of mathematical theories. Graph-Based Visual Saliency (GBVS) [72] was a bottom-up model that was based on graph theory to create activation maps from a raw image. The idea is that, if maps are implemented as Markov chain graphs, then the equilibrium (stable) distribution of the chain can be interpreted as activation and salience values over the locations of the map. The model contained two major parts—activation and normalization. Activation maps were based on feature channels that represented a set of meaningful locations according to a specific criterion, like information from human fixations. The graph-based Markovian approach found regions of dissimilarity that were computed in parallel to calculate mass at a region and distribute it among neighbouring regions. Such parallel processing at different regions leads to a single equilibrium distribution of mass. The normalisation stage integrated mass concentration over all activation maps in order to identify a set of salient locations. The same Markovian approach took place, and regions with the highest activation attracted mass. The model was made as a part of the GVBS Toolbox (refer to Appendix A for information on toolbox implementations) and provided a good example of a computationally driven implementation of the initial model.

Overall, these direct variations of the original model demonstrate that theoretical and practical augmentations can be made to the classic salience model while maintaining key components of its essential structure. These models all share preattentive feature processing, location-based feature extraction, and a focus on bottom-up attention, yet make their own contribution to new functionality. For most of these models, feature and location selection coincide, but we know that feature-based attention can exist independent of spatial locations during visual search [73]. To be accurate, identification of location should happen at a later stage and with fewer locations based on the processed relevant features.

### 4.2. Biologically Driven Models

Most computational models will claim some degree of biological plausibility, and the differences are often in the level of granularity they choose for their model. Some advances in salience modelling, however, have made a point to focus on the neural underpinnings of some portion of the processing stream. In this subsection, we look at four biologically-inspired models that share a similar architecture with the original model and focus on attaining a high level of biological validity of visual salience processes.

Li [74] introduced a theory of salience based on the independent sensitivity of V1 cells to specific features, and this was implemented in a bottom-up salience model by Koene and Li [75]. The main idea was that the features were not summed across channels to identify the most salient location as in the Itti and Koch model, but salience was identified at the location to which a specific V1 cell activated the most to as compared to other V1 cells. Moreover, the model implemented conjunctive cells in V1 that were sensitive to combinations of motion and orientation (MO) and colour and orientation (CO) [76,77]. This involved V1 neurons with receptive fields sensitive to orientation (O), motion (M), colour (C), colour and orientation (CO), and motion and orientation (MO). These neurons were connected horizontally, and a mutual inhibition mechanism was applied to neurons that activated to uniform features. Receptive fields with the highest activation thus represented a salient location. The model resulted in lower reaction times to targets with combined features as compared to targets with independent features processed in a parallel manner. Overall, the model had a strong theoretical and neural foundation and matched the simpler feature processing in striate structures [78,79].

Salience models frequently use multiple maps for features and scales (a total of 42 in the original Salience model), but this adds much redundant information. Park and colleagues [80] focused on reducing redundancy of retinal cells by adding a new pre-processing stage simulating LGN [81]. Input from natural coloured scenes created four feature maps for edges, symmetry, and red-green (RG) and blue-yellow (BY) colour opponency. Redundancy was reduced by applying an independent component analysis on the feature maps. An unsupervised learning approach was then used to assess the optimal weights in each feature map in order to combine them into an optimal salience map. The proposed model provided an alternative approach with a strong neural basis to the classic bottom-up salience model, though it did not take into account important characteristics of vision, like IOR.

The flat, two-dimensional maps of most salience models are a useful simplification of retinal input, but they do not account for biological features such as retinal sampling and magnification in the visual cortex nor consider the covert visual angle included in visual processing. The model by Aboudib and colleagues [82] differed from the Itti and Koch model in replacing Gaussian subsampling and across-scale reductions of the input image with kernel-based Gabor filtering and receptive field emulations that mimicked biological cortical magnification. This filtering approach produced a total of 9 feature maps per image instead of the initial 42 in the Itti and Koch model and lead to a generation of 250 most salient locations chosen based on a WTA/IOR process. The model computed input layers such as cone receptor or retinal ganglion cell layers, going upward to areas LGN, V1, etc. The model also allowed feature layers to be stacked onto each other before WTA/IOR took place, thus providing a way to mimic the feed-forward stream in the visual system.

As processing progresses along the ventral pathway, neuron receptive fields get larger and more specific to complex patterns. Hamker [83] proposed that FEF, based on Duncan’s theory on adaptive neural coding [84] might project complex templates backwards as a source of top-down attention. Specifically, the model implemented task-oriented feature-based attention in V4/TEO. It included top-down parameters into a bottom-up architecture in its feature processing stage, and contained ‘match detection units’ responsible for object detection in natural scenes. Processing used a population coding approach [85] to generate expectations based on prior knowledge, and thus, refresh the salience information of each feature based on top-down influences. The structure included several components: the early visual component for feature and conspicuity maps based on channels of colour opponency (two maps: RG, BY), intensities, orientations. and spatial resolutions; the V4 component, which coded the approximate salient stimuli locations; the TE component, which received pooled input from V4 in the form of feature conspicuity and location and resulted in several V4 features combining into one at TE; the FEF perceptual map, which integrated V4 and TE data across all five channels; and the FEF decision map, where several most salient locations competed for the highest saliency. A final IOR mechanism made sure that the model searched for objects in novel locations.

### 4.3. Top-Down Contributions

As mentioned earlier in Section 3, the Itti and Koch model did allow for basic top-down control through weighting of the different bottom-up features. Parameters could be set or learned that gave priority to colour or edges, for example, and these features would be weighted more strongly in the merged conspicuity map. The primary purpose of the model, however, was a demonstration of bottom-up salience, and it was left to future models to incorporate realistic aspects of top-down attention. To speak of a top-down contribution, however, we have to consider the attentional state of viewer and understand the intentions as defined by the viewing task [86]. Although task instructions are not the only manner of top-down influence, it is an established method of manipulating it experimentally. Although there have been many such contributions, we have chosen three models as exemplary.

Salience models are not restricted to visual search, but this specific task holds a prominent place in the literature. Jeremy Wolfe focused specifically on search instead of viewing without a specified task, and in doing so extended feature integration theory [15]. His Guided Search [87] model allowed searching for a target among a set of distractors and considered the bottleneck between visual input and object recognition. The model suggested that top-down information may be used to facilitate the search process by emphasizing bottom-up features of a target. The proposed architecture was divided into a preattentive stage, where a feature map distinguished targets from distractors using parallel processing, and a serial process stage, where each potential target was processed one by one using information from the first stage until the true target was found. Wolfe’s model was based on several theories of visual attention [15,88,89]. There have been several revisions of the model [50,67,90], with Guided search 4.0 perhaps undergoing the largest changes, as it represented visual guidance as an independent control mechanism, whereas in earlier versions, the preattentive stage played the role of a guidance mechanism. The mechanism proposed was a guidance activation map, which contained a weighted sum of bottom-up and top-down activations. Wolfe and Horowitz [59] further addressed the issue of identifying attributes that guide attention and provide convincing arguments for the existence of a guiding representation. Attentional guidance is also affected by previous experience and may manifest in inhibiting mechanisms, such as IOR [9,10,11], or in facilitation of the search for relevant stimuli [91,92].

The Feature Gate model [93] was created in an attempt to understand specific mechanisms of visual attention like parallel and serial search, inhibition of distractors, bottom-up feature selection, split attention, and feature contrast variations. The model used a hierarchical architecture consisting of a network of spatial maps containing attentional gates that controlled the signals sent to higher levels of processing. The decision to pass a signal was based on the usual bottom-up features but also on top-down characteristics that separated relevant features from non-relevant ones. Activations for each location were calculated separately for bottom-up and top-down information and then summed to form a general activation map of locations. Gates with the highest location activation values were allowed to pass the signal to the next level. The structure of the Feature Gate model was similar to the structural organization of the visual cortex, with the lowest map corresponding to area V1, containing small receptive fields sensitive to specific features, and the highest map similar to higher layers of the cortex, with large receptive fields sensitive to complex features. The model also implemented an inhibiting mechanism that prevented distractions, which helped differentiate relevant and non-relevant signals, which is similar to the biological mechanism of lateral inhibition [94].

Generative models, often based on Bayesian probability, have the added benefit of being able to simulate response distributions to new unseen exemplars. Rao and colleagues [95], for example, introduced two such probabilistic models of visual attention that included top-down influence on salience. The first model was based on reproducing of overt shifts of attention in visual search using salience maps of natural scenes. The most salient target location was defined using a probabilistic Boltzmann distribution algorithm [96,97] as a weighted average value of all scene locations with regards to the weight of the most salient location. Salience maps were generated through bottom-up and top-down parameters with reference to both spatial and target representations. The second model was based on predictive coding. It was a generative model of image representation used to study response properties in the visual cortex [98] and mimicked feedforward and feedback mechanisms of the ventral and dorsal pathways. This model demonstrated how visual attention is globally organised throughout various brain structures by predicting probable consequences of damage to different areas of the system. Both models shared a common probabilistic approach, but the second model added an extra focus on shifts of covert attention.

Maps that combine top-down and bottom-up information may more correctly be referred to as a priority map [17]. Though these models do not address all of the aspects of visual attention, each of them has made its own contribution to the field, with the Guided Search model focusing on strong cognitive hypotheses, the Feature Gate model imitating the overall structure of the visual cortex, and the generative nature of Rao’s probabilistic models. Hence, these models may be used as a basis for future models to further investigate biological visual attention processes.

### 4.4. The ‘What’ and ‘Where’ Models

In 1983, Mishkin, Ungerleider, and Macko [99] proposed a theory of two separate cortical pathways existing for object processing and spatial location processing and this was later elaborated by Milner and Goodale [23] as the ‘Two Streams hypothesis’. According to the theory, visual areas are hierarchically organized into two separate processing streams running ventrally and dorsally and are responsible for object (the ‘what’ stream) and spatial location (the ‘where’ stream) processing, respectively [24]. Here, we discuss two models of visual salience that take the separate processing streams theory into account.

Models of object recognition need to consider properties of perception such as object invariance. Rybak and colleagues [100] proposed just such a model that was invariant to scale changes and geometric transformations, such as rotations and translations. It modelled visual processing through three subsystems: a low level subsystem mimicking V1 neurons and responsible for edge detection; an intermediate level that used frames of reference to translate basic features into second order features insensitive to transformations; and a high level module that was based on the cooperation of the dorsal and ventral streams for sensory and motor memory accordingly. This final layer was further capable of operating in three task-modes: memorization, search and recognition. The model was tested and performed well on both complex scene and facial recognition tasks.

Likewise, the Neurodynamical Model [101] implemented both object-based and space-based streams [23,102] of visual attention with natural scenes as input. It was able to model increased receptive field sizes in higher layers in the visual cortex and also the top-down modulation (reduction) of receptive field size in complex scenes as compared to plain backgrounds [103]. The model comprised five modules, structured in a way that resembled the dorsal and ventral pathways and connected by feedforward and feedback projections. A short-term memory block was used to integrate top-down bias via pathways simulating the prefrontal cortex. The model was able to simulate fMRI signals using pooling of neurons in a specific area of the cortex with regards to spatial and temporal parameters and demonstrated dynamic interactions between the dorsal and ventral pathways.

While the independence of the dual streams may be over exaggerated [104], the underlying premise of the pathways remains [105]. The idea of parallel pathways for different aspects of visual processing should also have an appeal for modelling, where partitioning of functionality offers both ease of development and greater explanatory value. These models show the way forward for providing a benefit for including the neuronal mechanisms underlying the separate avenues for visual attention.

### 4.5. Object Models

An interesting approach to the modelling of visual attention is one based on object rather than feature salience. Object detection models in natural vision have their own rich literature [106,107,108] but we focus on two such models that combine objects with the Itti and Koch bottom-up architecture as one of the steps in the overall structure.

The ‘fingers of instantiation’ (FINST) theory by Pylyshyn [109,110] suggests that the visual system is capable of tracking a limited number of salient locations over time and across multiple fixations. The NAVIS system [111] comprised a bottom-up salience component, a top-down attentional control component, and a behavioral component divided into two stages. The first stage was similar to the Itti and Koch model, though it produced several salient locations based on the idea of FINST. The second stage analysed the locations chosen during the first stage and chose one focus for overt attention. The aim of the double-stage selection in the model was to combine theories of early and late selection into one system.

The model implemented feature maps for edges, oriented areas, and colours feeding into conspicuity maps of symmetry, eccentricity, and colour contrast, respectively. The derived maps were then combined into one bottom-up salience map, which was updated by top-down mechanisms marking locations for increased relevance. Dynamic neural fields [112] were implemented as an inhibiting mechanism, and this was based on two approaches for separate local and global inhibition. Inhibition of return in this model was applied globally to objects rather than salient points in space, which is consistent with Tipper’s [113] hypothesis of inhibiting mechanisms in humans.

Sun and Fisher [114] implemented a model based on object-based visual attention but incorporated components of multiple cognitive theories and studies of vision, such as the Integrated Competition Theory [115], the salience model of bottom-up attention [1,5], theories of top-down and bottom-up attention interaction, object and feature based salience, and others. The model suggested that competition for visual attention takes place both within an object and between objects and consisted of two main components. The first component was responsible for object salience and grouping and followed a similar strategy to other pre-attentive salience maps. The second component represented a hierarchical approach to attentional shifts. The first stage followed a similar strategy to other pre-attentive salience maps, and the second stage included hierarchical selection from the winners of the first stage from coarse to fine scales. This approach provided an integration of attentional selection from spatial locations, features, and feature conjunctions. The process took place between and within groupings across scales, and groupings at the final level marked a salient location.

Object recognition is a key component in vision processing. For example, the space between objects may be a key factor in spatial attention, search and reading [116]. Top-down factors, such as visual context and relevant task, are also important for object recognition, as they further facilitate the process of finding objects in certain contexts [117]. Object-based models might also provide some understanding of the interaction between feedforward and feedback mechanisms in the visual system, since such an interaction is considered important for the identification of the location of an object (for an example of such a model, see reference [118]). The feedback network could identify the location of objects, whereas the feedforward mechanism is responsible for the detection of shapes present in the visual field. The initial Itti and Koch model focused mainly on the spatial location of salient features, with object identification being a consequence of salient location detection, whereas object-based models deliberately concentrated on object salience by combining bottom-up and top-down approaches.

### 4.6. Computationally-Driven Models

Salience models have been a popular approach in psychology and neuroscience but also in applied fields like engineering and computer science where machine vision has shared many approaches with vision science. Linear combinations, normalisation algorithms, and Gaussian functions were instruments that helped the original model imitate bottom-up processes that take place in the visual cortex. Since then, many models that used computationally valid tools to predict visual salience have been proposed, and this subsection is dedicated to examples that have contributed to both applied and fundamental research.

The Selective Tuning model [119] shared a number of features with the Itti and Koch approach and was based on a hierarchical pyramid that initialised weights from a feedforward pass based on image input, followed by multiple feedback passes to tune selection. The selective tuning mechanism involved inhibition that behaved differently for spatial selection and feature selection. Spatial selection pruned irrelevant pyramid connections while feature selection inhibited components responsible for the computation of irrelevant features. After the inhibitory processes, a WTA mechanism was activated at the top layer of the hierarchy to determine a globally salient element. The model was one of the early implementations of feedback mechanisms as the signal was then sent back to the lower layers through direct connections, allowing the globally salient unit to influence lower level units, recursively identifying locally salient units at every level of the hierarchy.

Information theory in computer science and mathematics tries to quantify the value of any information by its ability to resolve uncertainty. Applying this idea to vision, we can think of fixation allocation as an extension of the question of how best choose locations that will maximize information gain. One example of this approach was AIM [120] that derived a self-information criterion from a natural image. An independent component analysis (ICA) was applied to patches of 7 × 7 pixels that were randomly selected from each image during the independent feature extraction stage. The likelihood per patch was then estimated and joined into a general likelihood for the entire image. The joint likelihood underwent Shannon’s self-information measure [121], after which a salience map was created for the input image. In general, the model represented an information maximization approach to a set of images. Although it performed better than the Itti and Koch model based on the area under the ROC curve score, it lacked both the cognitive foundations, like the feature integration theory, and the biological mechanisms like WTA and IOR. The model did provide a strong computational background, was based on the idea of sparse coding [122], and was an effective functional implementation directed at computer and robotic vision.

Information gain is also suitable for coding top-down information, as, for example, the Schill [123] model based on the Dempster-Shafer belief theory [124]. The model contained three basic levels—a neural network preprocessing stage that analysed information extracted from the scene, a top-down component based on the Dempster-Shafer belief theory for uncertain reasoning that applied to information from the preprocessing stage, and a control component that predicted fixations that would maximize information gain. This architecture would constantly update retrieved information from a scene to predict the most promising fixation locations. Systems based on information gain and entropy have an interesting theoretical implication in that IOR is no longer needed to prevent repeat fixations since the information gain at these locations is automatically reduced.

A creative approach to visual attention modelling was based on Bayes’ Theory. Torralba and colleagues [125] suggested using a Bayesian architecture, where attention was driven by global scene context. The authors proposed a contextual guidance model of attention based on two parallel pathways: one pathway for local feature salience, another for global feature processing that took scene characteristics into account. The salience pathway encoded each location independently, resulting in a bottom-up salience map. The global pathway extracted global features from the entire image filtered through six orientations and four scales. The result of the filtering was represented by a vector of 64 principal components. A ‘scene prior’ mechanism was then added based on the given task, and contextual modulation was applied to the bottom-up salience map, which resulted in a final context-sensitive, task-driven salience map. The global pathway made sure that most probable target locations were activated and that task-irrelevant salient locations were eliminated. The proposed model made accurate predictions of human visual behaviours based on a given task. It combined several theoretical approaches to visual attention, such as bottom-up salience and the effect of context on visual search.

Bayesian approaches are well suited for implementing context and task as the probability weights can shift based on evidence or prior knowledge. The SUN model [32], for example, regarded salience as differing based on the task being performed. In free-viewing, salience was based solely on bottom-up features, but with a specific task like target search, salience was based on the available knowledge about the visual features of the target class. The computation of the target probability was calculated per pixel and included bottom-up parameters, as well as visual appearance and location of a stimulus. Prior knowledge for the model was obtained using a set of images from the LabelMe dataset [126] which include prelabelled information on object location and identity. Testing of the model showed that it was able to predict human-like fixations, and even made mistakes similar to those made by people.

A simple but novel approach to salience modelling that addresses the role of foreground and background segmentation in visual attention [127] was proposed by Zhang and Sclaroff [128]. They used the Boolean map theory of visual attention [129] as a basis for their Boolean Map–based Salience model (BMS). Given an input image, BMS produced a set of Boolean maps based on binary figure-ground segregation according to randomly selected feature channels. The Boolean maps were then used to compute attention maps using Gestalt, figure-ground segregation principles to discover connected regions of the image. Normalised attention maps were then linearly combined into a single full-resolution salience map ready for object detection tasks or for the prediction of fixation locations. Despite its simple organisation, the BMS model maintained high levels of performance on several benchmark eyetracking datasets (see Appendix B).

The line between a strictly computational model and a cognitively plausible model is very much open to interpretation. One particular standout that has left a mark on approaches was a model proposed by Judd and colleagues [130]. It used a support vector machine (SVM) classifier method to learn how to estimate salience directly from human data. In the model, salient points on an image were defined by three layers of low-level features, which were then used to train the model. They also included ‘mid-level’ features such as automatic horizon line detectors and high-level features like face, people, and car detectors. The result was a model that was attracted to the same locations that attracted human fixations.

As seen from just several examples of computationally based models, there exist multiple approaches to mimicking visual salience with the help of computational tools. While computer vision and computational models of vision may have different goals, they have shared many algorithmic approaches over the years. Even computational approaches that are less biologically plausible, like ideal Bayesian observers [131], can be used to highlight the potential efficiencies of human search. Applied machine vision approaches do have an understandable focus on salience prediction through bottom-up salience since much top-down information would require some degree of common-sense knowledge.

## 5. Deep Learning Classifiers

In recent years, there has been a movement toward complex deep hierarchical structures for predicting salience in natural images [132,133]. This is because deep learning algorithms have been shown to be extremely accurate tools for modelling high levels of abstraction [134], such as vision and speech processes. They can also model human visual attention by drastically narrowing the gap in accuracy between model and human spatial predictions [135]. A neural network represents a set of elements called neurons, which are most commonly structured in the form of layers. Artificial neurons in neural networks are computationally similar to biological neurons, and early attempts to create neural networks were inspired by the human brain [136]. A biological network contains a collection of neurons that are electrically or chemically excited nerve cells and stores, processes, and passes signals to other neurons using electrical and chemical impulses. Artificial neurons may be described as a mathematical model of biological neurons, and these represent the basic element in an artificial neural network. Each neuron may receive several inputs, which are summed up and weighted to provide an output upon reaching a certain threshold. The input neurons are activated by a sensory signal that triggers evidence accumulation and pass weights to the next neural layers. The main advantage of a neural network is that it is possible to engage many neurons in parallel in order to solve different problems.

Deep learning is a type of machine learning algorithm that uses a non-linear function for parallel information processing [137]. It uses multi-layered (hence ‘deep’) neural networks and management of the neural weights to solve complex tasks or to replicate natural phenomena. Each layer of a deep network represents different levels of abstraction and is used to extract certain features of the data, such as images or audio. Deep learning architectures have proven to be useful in different spheres, such as biology, chemistry, finances, business, physics, neuroscience, and many others [138]. In vision research and spatial salience prediction, deep neural networks have become the leaders among other approaches as seen in the MIT saliency benchmark [139].

The class of deep learning networks most frequently used in visual processing is convolutional neural networks (CNN). CNNs have a multi-layered structure, but instead of learning all the weights between the nodes of neighbouring layers, they learn only the values of convolutional kernels, or filters, which are applied to the entire layer during the convolution operation. CNNs also have pooling layers and activation layers in the form of a rectified linear unit (ReLU) in addition to convolutional layers. Convolutional layers are responsible for the detection of local feature conjunctions, whereas pooling layers combine similar features into global features. The activation unit (ReLU) may be compared to a biological action potential that determines whether a neuron will fire or not. After a series of multiple sets of convolution, pooling and activation take place, the output progresses to a final, fully connected layer. The overall hierarchical organization of a CNN is said to be similar to the hierarchical organization of the visual cortex [140].

The applications of CNNs in salience detection are extensive. Some of the examples of these applications are Deep Gaze 2 [141], which presents two models for fixation predictions and object recognition; EML-NET [142] for salience feature detection using a modular approach; and DeepFix [143], which is sensitive to semantic information at different scales while using large receptive fields for global context analysis. The scope of existing models in the field is so broad that it is impossible to cover all of them in this review, but their accuracy in predicting areas of spatial salience is without question [144,145,146,147,148,149].

A recent trend in deep learning is the use of Generative Adversarial Networks (GANs), originally proposed by Ian Goodfellow and colleagues [150]. Unlike CNNs, GANs are generative, meaning that they are able to learn distributions of complex data and generate sets of new data that resembles the learned set. The term adversarial refers to the competitive process that takes place inside GANS—these networks contain two components, a generative and an adverse model, that are responsible for the generation and discrimination of data, respectively. SalGAN [151] used this technique to produce salience maps of an input image. GANs can also use conditional switches to generate maps for different images (SalGan) or different tasks [152] as an effective but simplistic mechanism for top-down attention. Although these networks are generative, to date, they have only been used to generate spatial saliency predictions and not temporal distributions of individual fixations like the more cognitive algorithms.

Recurrent neural networks (RNNs) have mostly been used for the processing of sequential data, like speech, but they have also been adapted for salience. RNNs contain units that are sensitive to temporal changes [153], namely looped connections that allow the information to be stored some period of time by being copied and passed to the next node. Their dynamic nature has allowed RNNs to serve as the basis for salience detection architectures in videos, such as the spatiotemporal grounding of evidence based on top-down factors [154] or the flow-guided approach to object detection [155].

Static scene salience detection have also used RNNs. For example, the DSRCNN model [156] uses recurrent network to enhance contextual information learning. Moreover, RNNs combined with CNNs have shown improved performance at salience detection based on different scales [157], as simple CNNs have a fixed receptive field size. The behaviour of RNNs has also been linked to biological lateral inhibition, as shown by the deep spatial contextual long-term recurrent convolutional network (DSCLRCN) [158] for fixation prediction in natural scenes.

RNNs can hold information for a short period of time, but result in the ‘vanishing gradient’ problem, which means that memory of a feature or input decays relatively quickly. This has led to the development of another artificial structure called long short-term memory (LSTM) networks, which were intended as explicit memory for recurrent architectures [159]. LSTM networks are a popular approach to solving long-term dependency problems, such as large-scale speech recognition [160,161] or object and salience detection in video sequences [162]. LSTMs have also proven to be useful in combination with other architectures in fixation prediction [158,163,164] and represent another possible implementation to include interactions between salience and working memory.

**Figure 2:**
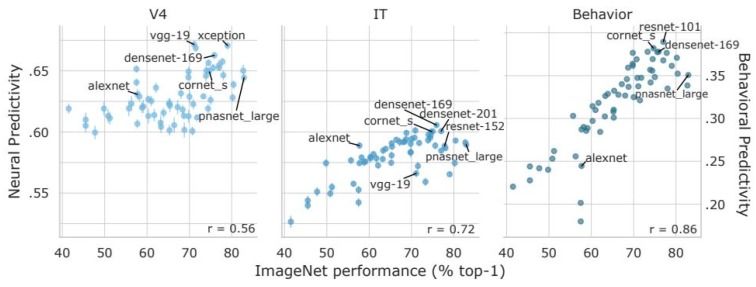
The performance of popular Deep Learning models based on their ability to match human V4 and inferior temporal (IT) regions, as well as human behavior performance. Adapted from “Brain-Score: which artificial neural network for object recognition is most brain-like?” by Schrimpf et al., 2018 [167], *BioRxiv*, 407007. Copyright 2018 by author. Reprinted with permission.

The leaps in accuracy for predicting areas of spatial salience have largely come from the deep learning approach of treating the image to salience map pipeline as a classification problem, which is a traditional strength of neural networks. However, to what degree can these classifiers be considered models in the computational cognitive neuroscience sense? The hierarchical organization of the visual cortex has certainly served as inspiration for deep learning models of visual attention, such as a deep belief network model for areas V1 and V2 [165]. Parallel processing is also an innate feature of deep learning neural networks, which allow them to process input in a way similar to the visual system. Another approach compares activation of artificial and biological networks at later layers of visual processing, such as area IT, to see how well deep learning architectures are able to match. For example, models as recent as Densenet-169 [166], created in 2016, have shown a trend for increased matching of human IT features in one of its layers [167]. Recently, further optimizations in classification accuracy for the ImageNet project [168] have produced models that have scored worse on brain benchmarks (Figure 2). Take, for example, popular models such as Inception [169] and PNASNet [170]. Despite their high performance on image recognition tasks, their predictivity of neural activity and human behavior falls short of anatomically simpler models such as CORnet [171].

## 6. Metrics and Evaluation

Despite the variety of approaches to salience modelling, there is still a lack of a standardized evaluation. Different metrics have been suggested to assess model performance, and the most frequently used sources is the MIT Saliency Benchmark [139]. It provides an open and free source for model assessment and benchmark data based on eight different evaluation metrics. The benchmark website includes most recent attempts at salience performance but also interesting performance baselines like the original Itti and Koch [1,5], central bias alone, and ‘infinite humans’ as a gold standard.

Most methods of assessment use location-based algorithms that measure the error between predicted and human fixations. For example, a simple correlation coefficient, or Pearson’s linear coefficient, measures the linear correlation or dependence between two salience maps. If the two maps are not correlated, the coefficient would be 0. One characteristic instrument of these metrics is the area under the Receiver Operating Characteristic curve (AUC ROC), which estimates the trade-off between true positive and false positive values at different discrimination thresholds by verifying that true positives are labelled before negative values [132,172]. One of the better AUC metrics and most commonly used for the evaluation task is the AUC-Judd [133,173]. The AUC-Judd interprets fixations as a classification task, where a pixel of the map may be either salient or not by applying a threshold over the intensity value of the saliency map [174]. Each salient pixel matching human fixations on the map is considered a true positive value, whereas salient pixels over non-fixation areas are classified as false positive values. The final AUC score is then calculated and plotted as a trade-off between true and false positive values. The highest possible score may be 1, whereas a 0.5 score is considered random. Another AUC approach is the AUC-Borji [175] metric that treats the salience map as a binary classifier. Positive and negative samples are differentiated through various thresholds. True positive values are then considered as values that exceed threshold at fixation locations, while false positive values are the values that exceed threshold at random locations. This approach differs from the AUC-Judd approach in that the threshold is set based on a fixed step size, and the false positive rate is calculated based on approximation of the Judd calculation. The shuffled AUC approach is also based on Ali Borji’s approach [175] but is additionally sensitive to centre bias. This metric shows whether a model has used centre bias for its predictions and imposes a penalty for this.

The Kullback-Leibler (KL) divergence [176] is a measure based on distribution. It evaluates the difference between the distribution of two different saliency maps, and measures information lost during the estimation of a fixation map with regards to the salience map. The lower the KL divergence value, the better is the approximation to ground truth in a salience map. However, the KL measure is extremely sensitive to zero values, which leads to a large penalty on the overall score if the predictions are deemed insufficient and sporadic.

Other options have been suggested such as the Earth Mover Distance (EMD) and the Normalized scanpath Salience (NSS), but these evaluation metrics have been adapted mostly for computer vision and based solely on spatial performance, whereas shifts of visual attention happen over time. Many of the classic salience models based off of Itti and Koch do use temporal layers to make predictions of fixation latencies, but even these show poor performance against human latency distributions [177]. Additionally, the existing metrics focus on the level of similarity between ground truth based on human data and the model data, but they do not take into account the cognitive basis underlying visual salience.

Although most models focus on the accuracy of classification, we can learn much about the differences between biological and artificial networks by finding examples where the predictions differ. Nguyen and colleagues [178] demonstrate this by generating images that DLNNs are unable to recognize but that a person would easily be able to. This demonstrates that an important factor in making human-like models is in the types of mistakes that humans and DLNNs tend to make. One of such models has already been mentioned in the current review [32], and it predicts human fixations at a decent level, while making similar mistakes to humans.

## 7. Conclusions

Roughly 20 years of salience model research has produced many excellent approaches that have improved our understanding of the visual processing stream. Various models have been created after the seminal Itti and Koch architecture, each bringing their own contributions, such as prediction accuracy, modelling additional tasks, the addition of object features and the inclusion of additional theoretical frameworks. Not every model had the same focus on all aspects of the five contributions we highlighted, i.e., either on the theoretical, neural, spatial, temporal, or computational aspects, but the overall progress in the field has been to push the boundaries in all of these directions.

One trend we would like to highlight, however, has been a recent shift away from visual attention modelling and toward fixation classification. The trending deep learning approaches are a very powerful computational tool; however, their current focus is not to explain visual processes but to reach state-of-the-art performance on spatial predictions. Due to this, important characteristics of visual attention, such as its temporal component and error production, have been widely ignored. The spectrum of existing powerful neural architectures, such as convolutional and recurrent neural networks, should provide a means of considering the temporal factors inherent to visual processes. We propose that an area of opportunity in the field is the exploration evaluation metrics that would account for all the above-mentioned aspects. Most current metrics are aimed at purely classification accuracy and focus on their ability to predict spatial locations, matching, and even surpassing human performance. While this trend has shown a great deal of success, the field of computational cognitive neuroscience should continue its goal of making biologically accurate and computationally powerful improvements to the Itti and Koch model for a deeper understanding of human visual attention processes. While we recognize the importance of purely computational approaches to visual attention directed at robotic vision, we suppose that the cognitive neuroscience community should shift its focus to a biologically and theoretically plausible approach at modelling visual attention. Instead of testing the limits of deep learning architectures, deep learning should be adopted as a means of testing cognitive theories and incorporating neural foundations of visual attention into the models. In particular, we suggest that testing our models against temporal distributions of attentional shifts, typical errors that humans make in visual processing and matching neural signatures of specific tasks will only improve the field going forward.

## Appendix A

The GVBS Toolbox [179] is available for open download at http://www.vision.caltech.edu/~harel/share/gbvs.php

There have also been computer toolboxes created based on the Itti and Koch architecture. The practical implementations of the initial model of bottom-up visual salience may be found in the Neuromorphic Visual Toolbox (NVT) [180] available at http://ilab.usc.edu/toolkit/ and the Saliency Toolbox [181] avalable at http://www.saliencytoolbox.net/.

## Appendix B

The benchmark datasets mentioned in the current article are the MIT Dataset [130], the Toronto Dataset [182], the Kootstra Dataset [183], the Cerf Dataset (FIFA dataset) [184], the ImgSal Dataset [185], and the CAT2000 dataset [186]
